# Non-steroidal anti-inflammatory drugs (NSAIDs) in cancer pain: A database analysis to determine recruitment feasibility for a clinical trial

**DOI:** 10.1177/02692163221122263

**Published:** 2022-09-14

**Authors:** Andrew J Page, Katie Spencer, Matthew R Mulvey, Barry JA Laird, Michael I Bennett

**Affiliations:** 1 Academic Unit of Palliative Care, Leeds Institute of Health Sciences, University of Leeds, Leeds, UK; 2 University of Leeds Faculty of Medicine and Health, Academic Unit of Health Economics, Leeds Institute of Health Sciences, Leeds, UK; 3 Edinburgh Cancer Research Centre, University of Edinburgh, Edinburgh, UK

**Keywords:** Cancer pain, anti-inflammatory agents, non-steroidal, bone neoplasms, palliative care

## Abstract

**Background:**

Insufficient evidence exists to support or refute use of NSAIDs for managing cancer pain. Palliative physicians support a placebo-controlled trial of NSAIDs as strong opioid adjuncts for cancer-induced bone pain as the most pragmatic design to benefit clinical practice.

**Aim:**

We aimed to determine patient numbers receiving palliative radiotherapy for cancer-induced bone pain, estimate the suitability of NSAID prescription and determine survival, guiding future trial feasibility.

**Design:**

A retrospective observational database analysis was undertaken using 5 years of routinely collected regional radiotherapy and healthcare data, filtered to achieve a cohort with cancer-induced bone pain. Demographics and survival were linked to available serology and co-morbidity data.

**Setting/participants:**

Data was sourced from the regional Leeds Cancer Centre, a tertiary care setting. Patients who underwent palliative single fraction 8 gray (Gy) radiotherapy treatment for cancer-induced bone pain were included, totalling 2411 over 5 years.

**Results:**

A mean of 478 patients received palliative radiotherapy for cancer-induced bone pain annually. Median age (IQR) was 70 (62–77); negatively skewed (−0.69). 65.3% died within 1 year of radiotherapy; 48.0% within 6 months. Age was not associated with survival on univariable analysis (HR 0.999 (95% CI 0.996–1.003)). Serology from 1063 patients (44.2%) were available; eGFR was ⩾60 mL/min/1.73 m^2^ in 83.0%. From available data (1352 pts; 51.6% of sample), 20.2% had a coded co-morbidity contra-indicating NSAIDs. Combining serology and co-morbidities, 68.5% could be considered for NSAID prescription.

**Conclusions:**

Patient numbers at a regional radiotherapy centre support the feasibility of trial recruitment. Available serology and co-morbidity data suggest two-thirds may be suitable for NSAID prescription.

**What is already known about the topic?**

- Cancer pain is common, extremely debilitating, and undertreated worldwide.
- We do not know if non-steroidal anti-inflammatory drugs (aka NSAIDs or “anti-inflammatories”) are effective in managing cancer pain of any type.
- To further scientific understanding, UK palliative care doctors advocate a pragmatic trial to determine the role, if any, of NSAIDs as opioid adjuncts for treating cancer-induced bone pain.

**What this paper adds**

- Numbers treated for cancer-induced bone pain at a single regional radiotherapy centre (478 per year) support the feasibility of trial recruitment.
- Considering eGFR and contraindicating co-morbidities, two-thirds could be suitable for NSAID prescription if proven efficacious.
- Suitability for NSAID prescription reduces with age, with the proportion unsuitable increasing in those over 65 years old.

**Implications for practice, theory, or policy**

- Recruitment to a future trial of NSAIDs in the management of cancer-induced bone pain appears feasible, particularly if multiple recruitment centres are used.
- Demonstrating feasibility allows the planning of a definitive clinical trial to determine the efficacy of NSAIDs in this patient group.
- Without a definitive clinical trial, the question remains: are effective analgesics being underutilised in cancer pain management, or are ineffective medications increasing the risk of side effects in an already co-morbid cancer population?

## Background

Pain remains a significant burden for people with cancer, reported in 55% receiving anti-cancer treatment and 66% with advanced disease.^1^ Pain negatively affects health-related quality of life, social activity, psychological health, and anti-cancer treatment adherence.^2^ Despite this, an estimated one-third of people with cancer have inadequate pain control.^3^ At the end of life, pain remains the principal fear amongst the wider UK public, a concern shared by doctors.^4^ Although multiple barriers to adequate pain management exist, fundamental questions concerning the efficacy of certain analgesics or analgesic combinations for patients with cancer remain.

Non-steroidal anti-inflammatory drugs (NSAIDs) are non-opioid analgesics used in the management of cancer pain. This is supported by the World Health Organisation (WHO) who advocate use of NSAIDs in mild-moderate cancer pain, as well as an opioid adjunct in more severe pain.^1^ However, Cochrane concludes “there remains no high-quality evidence to support or refute the use of NSAIDs alone or in combination with opioids for the three steps of the WHO cancer pain ladder.”^5^ Complicated by a significant side-effect profile including gastrointestinal, cardiovascular, and renal harms, determining if or when to prescribe NSAIDs for patients with cancer pain remains challenging.

Although current UK prescribing rates in cancer pain are unknown, a national survey of UK palliative care physicians suggested NSAIDs are being predominantly utilised as strong opioid adjuncts clinically.^6^ Consensus concerning efficacy was mixed, with most considering cancer-induced bone pain the ICD-11 pain type for which NSAIDs are most utilised in practice.^6^ Consequently, a randomised control trial to determine the efficacy of NSAIDs as strong opioid adjuncts for cancer-induced bone pain was considered the most pragmatic design to guide clinical practice.^6^

Previous research has identified cohorts with cancer-induced bone pain from those referred for palliative radiotherapy.^7^ Therefore, to determine trial feasibility, we aimed to determine patient numbers with cancer-induced bone pain treated at a regional radiotherapy centre in the UK, as well as determine survival and estimate the suitability of NSAID prescription in this cohort.

## Method

### Design

A retrospective observational database analysis was undertaken using 5 years (01/01/2016 – 05/02/2021) of routinely collected regional radiotherapy and healthcare data.

### Cohort

Retrospective data obtained from the regional Leeds Cancer Centre (Leeds Teaching Hospitals NHS Trust) was filtered and cleaned (Appendix 1) to identify a cancer-induced bone pain cohort. All were aged ⩾18 years and were treated with a single 8 Gy radiotherapy fraction to a bone specific treatment site (recorded as radiotherapy Z codes). Of note, emergency radiotherapy treatment codes (including those treated for metastatic spinal cord compression) were excluded during filtering (Appendix 1), to ensure those in whom other ICD-11 pain types predominate did not falsely inflate cohort estimates. For all steps used in data filtering see Appendix 1. For accuracy of cohort derivation, a random patient selection (50 included, 50 excluded) following filtering had their medical notes manually reviewed to confirm radiotherapy indication; 94% were appropriately included and 90% appropriately excluded.

### Data

Leeds Teaching Hospitals data concerning demographics (age, sex, postcode of residence), co-morbidities, preceding serology, subsequent radiotherapy, and survival was extracted. Due to expected mortality rates, the ethics committee agreed consent was not possible. However, patients with national data opt-out were excluded. Local and national governance approval was received.

### Analysis

Analyses were performed using IBM SPSS Statistics (v.26). Data was summarised descriptively as proportions for categorical variables. Medians, interquartile ranges, and skew were used for age and travel distance. A Cox proportional hazards model examined the association between age (continuous variable) and survival following radiotherapy. To estimate patient proportions suitable for an NSAID trial, a lack of contra-indicated co-morbidities (as per British National Formulary^8^) and an eGFR >60 mL/min/1.73 m^2^ were used.

## Results

Over 5 years, 2411 patients were treated with palliative radiotherapy for cancer-induced bone pain in Leeds. Five were excluded via national data opt-out. Median age (IQR) was 70 (62–77); negatively skewed (−0.69). More were male (58%). A mean (SD) of 478 (20.7) patients were treated annually. Median (IQR) distance travelled for radiotherapy was 18.2 (11.8–26.7) miles, positively skewed (4.95).

### Treatment site and subsequent radiotherapy

Over half were treated for spinal bone metastasis (53.3%). Of these, individual treatments to thoracic (31%) or lumbar (29%) regions were most represented; 22% received treatment to multiple spinal regions (e.g. ⩾2 of cervical, thoracic, lumbar or sacral). Other common sites included the pelvis (17.4%) and hip/femur (14.4%). A minority (30.1%) attended hospital for further radiotherapy on subsequent dates.

### Survival

Within 1 year of their first palliative radiotherapy for cancer-induced bone pain 65.3% of patients died, with 30.5% and 48.0% dying within 3 and 6 months respectively. Age was not associated with survival on univariable analysis (HR 0.999 (95% CI 0.996–1.003)).

### Serology and co-morbidity data

During the 6 months prior to radiotherapy, blood results from 1063 patients (44.2%) were available; eGFR was >90 mL/min/1.73 m^2^ in 500 (47.0%) and >60 mL/min/1.73 m^2^ in 882 (83.0%). Similarly, a minority had markers of impaired synthetic liver function (platelets <150 10^9^/L in 84 (7.9%); bilirubin ⩾21 in 36 (3.4%); INR ⩾ 1.2 in 218 (20.5%)), excluding hypoalbuminaemia (575 (54.1%)). Transaminases were within normal range for the majority (914 (86.0%)) or only mildly elevated (101 (9.95%) with alanine transaminase up to twice normal). The majority (875 (82.3%)) had haemoglobin levels of ⩾100 g/L.

Co-morbidity data was available for 1351 patients (56.1%), 1288 (95.3%) of whom had at least one recorded co-morbidity. Specifically, 273 (20.1%) had at least one co-morbidity contra-indicating NSAID use, of which 84.2% had ⩾1 cardiovascular co-morbidity (ischaemic heart disease, congestive cardiac failure, peripheral arterial disease).

**Figure 1.**
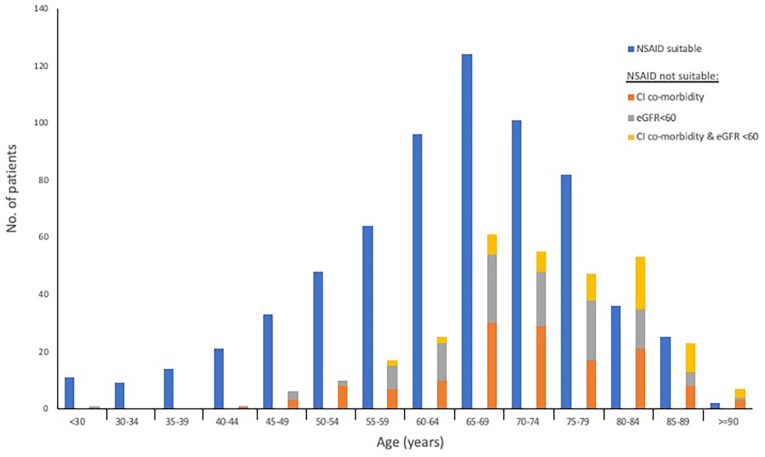
NSAID suitability by age group (*n* = 971). If not suitable, separated by an eGFR < 60, a contra-indicated comorbidity or both.

Combined serological and co-morbidity data was available for 971 patients (40.3%) [Figure 1]. Of this, 665 (68.5%) were considered suitable for NSAID prescription in a future trial (eGFR >60 mL/min/1.73 m^2^ and no contra-indicated co-morbidities).

## Discussion

To date, few exemplar studies demonstrate how to best conduct cancer pain trials with non-opioid drugs, such as NSAIDs.^5^ With under-recruitment, missing data and attrition limiting NSAID trial quality in cancer, estimates of incidence and recruitment figures will be the cornerstone of a methodologically rigorous trial.^5^ This study demonstrates a way of identifying a cohort with cancer-induced bone pain in sufficient numbers, supporting the feasibility of trial recruitment, particularly if undertaken from multiple sites. Despite challenges in palliative cohorts, a multi-centre model has precedent in recent drug trials.^7,9^

Despite being the most common cancer pain type, the exact incidence of cancer-induced bone pain remains uncertain.^10^ Combining palliative radiotherapy data in England with published proportions treated for cancer-induced bone pain, an annual incidence of 18,000 patients treated for cancer-induced bone pain can be estimated.^11,12^ This mirrors UK wide approximations.^13^ Additionally, as modern oncological treatment continues to improve metastatic cancer survival, often over several years, cancer-induced bone pain prevalence may be expected to increase more swiftly than its annual incidence.^10^ Considering extrapolation of these figures internationally, a significant population exists who would benefit from more definitive research.

With numbers tailored using available serological and co-morbidity data, we estimate two-thirds of patients in this cohort could be eligible for NSAID prescription. Analgesic prescription decisions are nuanced in cancer pain management, guided by factors including pain pathophysiology, severity, serology, co-morbidity, patient preference, drug interactions and the evidence base available. With so many factors underpinning the risk: benefit ratio that guides doctor-patient discussions and NSAID prescription decisions, our estimate may lack precision. A significant majority demonstrating an eGFR ⩾60 mL/min/1.73 m^2^ is reassuring, a more conservative cut-off compared to NICE NSAID contraindications (<30 mL/min/1.73 m^2^).^14^

One significant consideration in trial design concerns the clinical complexity of cancer-induced bone pain as a pain state, which can involve “a combination of background, spontaneous, and incident (movement-evoked) pain.”^15^ Europe-wide observational research suggests >85% with cancer-induced bone pain report some form of incident pain episodes.^16^ In view of such varied symptomatology, trial outcome measures, and sample size calculations will have to be carefully considered to ensure analgesic effects are not missed.

Missing co-morbidity and serology data remains the main limitation. Less data was available for patients living outside Leeds, therefore missing data could reflect the radiotherapy providers large geographical catchment. With most data derived from recent tertiary hospital admissions it is possible cohort co-morbidity could be overestimated if extrapolated, considering co-morbidity correlates with likelihood of inpatient hospital care.^17^ On the contrary, those with extensive co-morbidity may avoid inpatient admission via pragmatic advanced care planning. However, this argument is not supported by the relatively low rates of advanced care planning in UK hospital admissions.^18^ Equally, with a reliance on retrospective coded hospital data it is possible some available co-morbidity data may be inaccurate, incomplete or lack nuance. Our pragmatic approach was taken to ensure timely results that would guide future trials.

It is also of note that our filtering algorithm was imperfect in identifying those with cancer-induced bone pain from radiotherapy data. However, with accuracy estimates of at least 90% from cohort derivation, these small inaccuracies are unlikely to impact conclusions. Historically poor classification of cancer-related pain types means that separating specific pain syndromes in cancer is not possible from retrospective records. Only in recent years have the nuance of cancer-related pain types been more specifically defined, providing hope for future research in these cohorts.^19^

An estimated one quarter of patients with cancer-induced bone pain have a complete response to palliative radiotherapy, leaving a significant proportion with ongoing pain.^20^ Interestingly, certain cytokines are being theorised as predictors of radiotherapy response in cancer-induced bone pain.^21^ As medicine moves towards more targetted individualised care, robust biomarkers could help focus radiotherapy treatment on likely responders, with alternative treatments considered earlier in those less likely to respond.

Inequality in access to palliative radiotherapy based on a patient’s proximity to a radiotherapy centre is also well documented.^22^ This may be reflected in the skewed travel distance identified, although could reflect differing population density or demographics beyond city boundaries. Those more geographically isolated would likely benefit most from more definitive NSAID research.

Considering survival post radiotherapy, NSAIDs could provide sustained benefit for the majority with ongoing pain if proven efficacious. NSAID suitability by age group (Figure 1) correlates with NICE guidance, which identifies those aged ⩾65 years as being at increased risk of gastrointestinal, renal and cardiovascular harms.^14^ Trial recruitment will have to carefully consider NSAID suitability in those ⩾65 years, a dilemma mirroring clinical practice.

In practice, patients can be prescribed NSAIDs over weeks, months or even years.^5^ Cochrane identified only a single trial with a duration of >2 weeks, with data on NSAID adverse events deemed very low-quality in cancer pain.^5^ Consequently, no assertions regarding long term efficacy or risks can be made. If repeated exposure cumulatively increases the incidence of certain adverse events (e.g. gastrointestinal tract ulcers), shorter studies maybe falsely reassuring.^5,23^ Any extrapolation of NSAID side effects from chronic use in non-malignant disease requires caution.^6^ With attrition plaguing palliative care trials, it is unlikely a single trial design could determine both short and longer-term NSAID efficacy and side effects. Most palliative care physicians assess NSAID efficacy in clinical practice within 1 week of commencement, a duration which could be conveniently situated before radiotherapy.^6^ This design would be favourable, as opposed to treating with NSAIDs alongside radiotherapy as demonstrated in other drug trials.^7^

In conclusion, palliative physicians advocate a pragmatic trial to evaluate the benefit of NSAIDs as opioid adjuncts for cancer-induced bone pain.^6^ Our research demonstrates the feasibility of identifying a cohort of patients with cancer-induced bone pain in sufficient numbers to support a multi-centre randomised controlled trial. Extrapolated estimates of incidence, paired with available serology, co-morbidity and survival data support a role for NSAIDs in cancer-induced bone pain if proven efficacious.

## Supplemental Material

sj-pdf-1-pmj-10.1177_02692163221122263 – Supplemental material for Non-steroidal anti-inflammatory drugs (NSAIDs) in cancer pain: A database analysis to determine recruitment feasibility for a clinical trial Click here for additional data file. Supplemental material, sj-pdf-1-pmj-10.1177_02692163221122263 for Non-steroidal anti-inflammatory drugs (NSAIDs) in cancer pain: A database analysis to determine recruitment feasibility for a clinical trial by Andrew J Page, Katie Spencer, Matthew R Mulvey, Barry JA Laird and Michael I Bennett in Palliative Medicine

## References

[1] World Health Organisation (WHO). WHO guidelines for the pharmacological and radiotherapeutic management of cancer pain in adults and adolescents, https://www.who.int/publications/i/item/9789241550390 (2019, accessed August 2021). 30776210

[2] Scarborough B Smith CB . Optimal pain management for patients with cancer in the modern era. CA Cancer J Clin 2018; 68(3): 182–196. 29603142 10.3322/caac.21453 PMC5980731

[3] Greco MT Roberto A Corli O , et al. Quality of Cancer Pain Management: an update of a systematic review of undertreatment of patients with cancer. J Clin Oncol 2014; 32(36): 4149–4154. 25403222 10.1200/JCO.2014.56.0383

[4] British Medical Association. End-of-life care and physician-assisted dying – Public dialogue research, https://www.bma.org.uk/media/1417/bma-end-of-life-care-and-physician-assisted-dying-volume-two-report.pdf (2015, accessed August 2021).

[5] Derry S Wiffen PJ Moore RA , et al. Oral nonsteroidal anti-inflammatory drugs (NSAIDs) for cancer pain in adults. Cochrane Database Syst Rev 2017; 7(7): CD012638. 10.1002/14651858.CD012638.pub2 PMC6369931 28700091

[6] Page AJ Mulvey MR Bennett MI . Designing a clinical trial of non-steroidal anti-inflammatory drugs for cancer pain: a survey of UK palliative care physicians. BMJ Support Palliat Care 2020; bmjscare–2020. Published Online First 02 December 2020. 10.1136/bmjspcare-2020-002792 33268476

[7] Fallon M Hoskin PJ Colvin LA , et al. Randomized double-blind trial of pregabalin versus placebo in conjunction with palliative radiotherapy for cancer-induced bone pain. J Clin Oncol 2016; 34(6): 550–556. 26644535 10.1200/JCO.2015.63.8221 PMC5098845

[8] Joint Formulary Committee. British National Formulary (online). London: BMJ Publishing Group and Pharmaceutical Press, https://bnf.nice.org.uk (2021, accessed August 2021).

[9] LeBlanc TW Lodato JE Currow DC , et al. Overcoming recruitment challenges in palliative care clinical trials. J Oncol Pract 2013; 9(6): 277–282. 24130254 10.1200/JOP.2013.000996 PMC3825289

[10] Caraceni A Portenoy RK . An international survey of cancer pain characteristics and syndromes. IASP Task Force on Cancer Pain. International Association for the Study of Pain. Pain 1999; 82(3): 263–274. 10488677 10.1016/S0304-3959(99)00073-1

[11] National Cancer Registration and Analysis Service (NCRAS). Radiotherapy Dataset (RTDS) – Radiotherapy Delivery in England, https://www.cancerdata.nhs.uk/radiotherapy/dashboard (2021, accessed 18 March 2022].

[12] Spencer K Morris E Dugdale E , et al. 30 day mortality in adult palliative radiotherapy – A retrospective population based study of 14,972 treatment episodes. Radiother Oncol 2015; 115(2): 264–271. 25861831 10.1016/j.radonc.2015.03.023 PMC4504022

[13] Kane CM Hoskin P Bennett MI . Cancer induced bone pain. BMJ 2015; 350: h315. 10.1136/bmj.h315 25633978

[14] NICE Clinical Knowledge Summaries. Scenario: NSAIDs – prescribing issues https://cks.nice.org.uk/topics/nsaids-prescribing-issues/management/nsaids-prescribing-issues/ (2020, accessed December).

[15] Falk S Dickenson AH . Pain and nociception: Mechanisms of cancer-induced bone pain. J Clin Oncol 2014; 32(16): 1647–1654. 24799469 10.1200/JCO.2013.51.7219

[16] Davies A Buchanan A Zeppetella G , et al. Breakthrough cancer pain: an observational study of 1000 European oncology patients. J Pain Symptom Manag 2013; 46: 619–628. 10.1016/j.jpainsymman.2012.12.009 23523361

[17] Wang HH Wang JJ Lawson KD , et al. Relationships of multimorbidity and income with hospital admissions in 3 health care systems. Ann Fam Med 2015; 13(2): 164–167. 25755038 10.1370/afm.1757 PMC4369606

[18] Knight T Malyon A Fritz Z , et al. Advance care planning in patients referred to hospital for acute medical care: results of a national day of care survey. EClinicalMedicine 2020; 19: 100235. 32055788 10.1016/j.eclinm.2019.12.005 PMC7005412

[19] Bennett MI Kaasa S Barke A , et al. IASP taskforce for the classification of Chronic Pain. The IASP classification of chronic pain for ICD-11: chronic cancer-related pain. Pain 2019; 160(1): 38–44. 30586069 10.1097/j.pain.0000000000001363

[20] Chow E Zeng L Salvo N , et al. Update on the systematic review of palliative radiotherapy trials for bone metastases. Clin Oncol 2012; 24(2): 112–124. 10.1016/j.clon.2011.11.004 22130630

[21] MacLeod K Laird BJA Carragher NO , et al. Predicting response to radiotherapy in cancer-induced bone pain: cytokines as a potential biomarker? Clin Oncol 2020; 32(10): e203–e208. 10.1016/j.clon.2020.03.010 32284199

[22] Chand CP Greenley S Macleod U , et al. Geographical distance and reduced access to palliative radiotherapy: systematic review and meta-analysis. BMJ Support Palliat Care 2022; bmjscare–2021. Published Online First 15 March 2022. 10.1136/bmjspcare-2021-003356 35292512

[23] Magee DJ Jhanji S Poulogiannis G , et al. Nonsteroidal anti-inflammatory drugs and pain in cancer patients: a systematic review and reappraisal of the evidence. Br J Anaesth 2019; 123(2): e412–e423. 10.1016/j.bja.2019.02.028 PMC6676054 31122736

