# Supplementary material for: Non-steroidal anti-inflammatory drugs (NSAIDs) in cancer pain: A database analysis to determine recruitment feasibility for a clinical trial
Source: Palliat Med. 2022 Sep 14;36(9):1440–5. doi: 10.1177/02692163221122263 (PMC9596945; doi:10.1177/02692163221122263)
Supplement: sj-pdf-1-pmj-10.1177_02692163221122263 – Supplemental material for Non-steroidal anti-inflammatory drugs (NSAIDs) in cancer pain: A database analysis to determine recruitment feasibility for a clinical trial [file sj-pdf-1-pmj-10.1177_02692163221122263.pdf]

Appendix 1

Steps for data filtering at radiotherapy department at LTHT:

1. Filter 8Gy single # in over 18yrs
2. Exclude emergency treatments
3. Exclude electron treatments
4. Exclude TreatmentRegion coded with an O (keep those coded with P)
5. Exclude a list of specific Z codes – see below.
    a. Included/ remaining Z codes listed below also
6. Exclude any Site_Name that contains "prostate" of "bladder"
7. Exclude specific 'descriptions of treatment site' – see below.
8. Include only those given with palliative intent

Excluded Z codes:
Z01.4, Z01.9, Z05.2, Z05.8, Z08.1, Z09.4, Z15.5, Z15.6, Z15.8, Z15.9, Z16.1, Z20.1, Z21.9, Z22.4, Z22.9, Z23.9, Z24.1, Z24.6, Z24.7, Z25.1, Z25.5, Z25.6, Z25.8, Z26.1, Z26.9, Z27.1, Z27.2, Z29.1, Z29.2, Z29.8, Z30.1, Z31.1, Z31.3, Z31.8, Z33.1, Z41.1, Z41.3, Z42.1, Z42.9, Z43.3, Z43.6, Z44.3, Z44.9, Z45.0, Z45.1, Z45.9, Z46.3, Z47.1, Z47.2, Z47.3, Z47.5, Z48.1, Z49.1, Z49.2, Z49.4, Z49.5, Z50.1, Z50.2, Z50.4, Z52.1, Z52.8, Z52.9, Z53.2, Z54.2, Z54.8, Z54.9, Z57.2, Z57.8, Z57.9, Z60.1, Z60.3, Z60.9, Z61.3, Z61.5, Z61.8, Z61.9, Z63.8, Z63.9, Z89.3, Z90.1, Z90.3, Z92.3, Z92.4, Z92.5, Z92.6

Remaining/ included Z codes:
Z06.1, Z06.2, Z06.3, Z06.4, Z06.5, Z06.8, Z06.9, Z07.1, Z07.2, Z07.3, Z08.3, Z09.2, Z09.6, Z09.8, Z12.2, Z23.1, Z42.2, Z63.4, Z64.3, Z65.1, Z66.1, Z66.2, Z66.3, Z66.4, Z66.5, Z66.8, Z66.9, Z67.2, Z67.3, Z67.4, Z67.5, Z67.6, Z68.1, Z68.5, Z68.8, Z68.9, Z69.1, Z69.2, Z69.3, Z69.4, Z69.8, Z69.9, Z71.9, Z72.8, Z72.9, Z74.2, Z74.3, Z74.4, Z74.5, Z74.6, Z74.8, Z74.9, Z75.1, Z75.2, Z75.3, Z75.4, Z75.5, Z75.6, Z75.8, Z75.9, Z76.1, Z76.2, Z76.3, Z76.4, Z76.5, Z76.8, Z76.9, Z77.2, Z77.4, Z77.8, Z77.9, Z78.1, Z78.3, Z78.6, Z78.9, Z79.8, Z81.4, Z81.5, Z82.9, Z84.1, Z84.2, Z84.3, Z84.6, Z84.8, Z84.9, Z85.6, Z89.1, Z89.2, Z89.9, Z90.2, Z90.4, Z90.5, Z92.2

Excluded 'descriptions of treatment site':
Malignant neoplasm of bladder, unspecified
Malignant neoplasm of cervix uteri, unsp
Malignant neoplasm of vulva, unspecified
Malignant neoplasm of endometrium
Diffuse non-Hodgkin's lymphoma, unspecified
Follicular non-Hodgkin's unspecified lymphoma
Kaposi's sarcoma of soft tissue
Malignant melanoma of skin, unsp
Malignant neoplasm of caecum
Malignant neoplasm of endometrium
Malignant neoplasm of ovary
Malignant neoplasm of penis, unspecified
Malignant neoplasm of rectosigmoid junction
Malignant neoplasm of rectum

Malignant neoplasm of uterus, part unspecified
Malignant neoplasm of vagina
